# Factors That Can Affect the External Validity of Randomised Controlled Trials

**DOI:** 10.1371/journal.pctr.0010009

**Published:** 2006-05-19

**Authors:** Peter M Rothwell

Randomised controlled trials (RCTs) must be internally valid (i.e., design and conduct must eliminate the possibility of bias), but to be clinically useful, the result must also be relevant to a definable group of patients in a particular clinical setting (i.e., they must be externally valid). Lack of external validity is the most frequent criticism by clinicians of RCTs, systematic reviews, and guidelines, and is one explanation for the widespread underuse in routine practice of many treatments that have been shown to be beneficial in trials and are recommended in guidelines [1]. Yet medical journals, funding agencies, ethics committees, the pharmaceutical industry, and governmental regulators seem to give external validity a low priority. Admittedly, whereas the determinants of internal validity are intuitive and can generally be worked out from first principles, understanding of the determinants of the external validity of an RCT requires clinical rather than statistical expertise, and often depends on a detailed understanding of the particular clinical condition under study and its management in routine clinical practice. However, reliable judgments about the external validity of RCTs are essential if treatments are to be used correctly in as many patients as possible in routine clinical practice.

The results of RCTs or systematic reviews will never be relevant to all patients and all settings, but they should be designed and reported in a way that allows clinicians to judge to whom the results can reasonably be applied. Table 1 lists some of the important potential determinants of external validity, each of which is reviewed briefly below. Many of the considerations will only be relevant in certain types of trials, for certain interventions, or in certain clinical settings, but they can each sometimes undermine external validity. Moreover, the list is not exhaustive and requires more detailed annotation and explanation than is possible in this short review.

Some of the issues that determine external validity are relevant to the distinction between pragmatic trials and explanatory trials [2], but it would be wrong to assume that pragmatic trials necessarily have greater external validity than explanatory trials. For example, broad eligibility criteria, limited collection of baseline data, and inclusion of centres with a range of expertise and differing patient populations have many advantages, but they can also make it very difficult to generalise the overall average effect of treatment to a particular clinical setting.

## The Setting of the Trial

**Figure 1:**
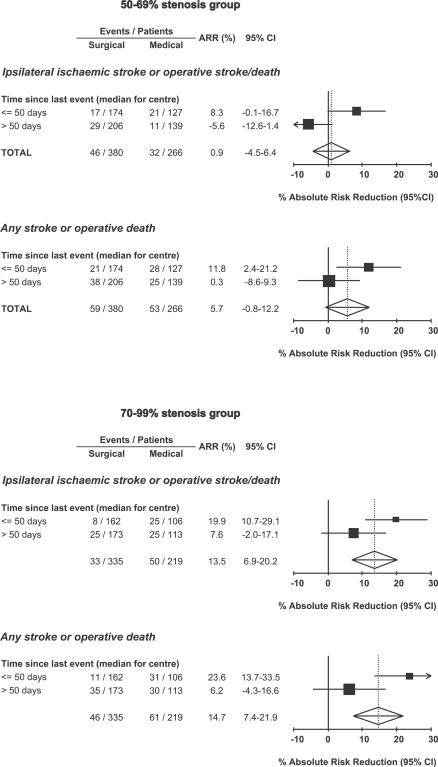
The Absolute Reductions in the Five-Year Risks of Ipsilateral Ischaemic Stroke (Top) and Any Stroke or Death (Bottom) with Surgery in European Carotid Surgery Trial Centres in Which the Median Delay from Last Symptomatic Event to Randomisation Was Less than or Equal to 50 Days (Fast Centres) Compared with Centres with a Longer Delay (Slow Centres) Data are shown separately for patients with moderate (50%–69%) and severe (70%–99%) carotid stenosis.

A detailed understanding of the setting in which a trial is performed, including any peculiarities of the health-care system in particular countries, can be essential in judging external validity. The potential impact of differences between health-care systems is illustrated by the analysis of the results of the European Carotid Surgery Trial (ECST) [3], an RCT of endarterectomy for recently symptomatic carotid stenosis, in Figure 1. National differences in the speed with which patients were investigated, with a median delay from last symptoms to randomisation of greater than two months in the United Kingdom (slow centres) compared with three weeks in Belgium and Holland (fast centres), resulted in very different treatment effects in these different health-care systems—due to the shortness of the time window for effective prevention of stroke. Similar differences in performance between health-care systems will exist for other conditions, and there is, of course, the broader issue of how trials done in the developed world apply in the developing world. Moreover, other differences between countries in the methods of diagnosis and management of disease—which can be substantial—or important racial differences in pathology and natural history of disease also affect the external validity of RCTs. A good example is the heterogeneity of results of trials of bacilli calmette guerin vaccination in prevention of tuberculosis, with a progressive loss of efficacy (*p* < 0.0001) and with decreasing latitude [4].

How centres and clinicians were selected to participate in trials is seldom reported, but can also have important implications for external validity. For example, the Asymptomatic Carotid Artery Study (ACAS) trial of endarterectomy for asymptomatic carotid stenosis only accepted surgeons with an excellent safety record, rejecting 40% of applicants initially, and subsequently barring from further participation those who had adverse operative outcomes in the trial. The benefit from surgery in ACAS was due in major part to the consequently low operative risk [5]. A meta-analysis of 46 surgical case series that published operative risks during the five years after ACAS found operative mortality to be eight times higher and the risk of stroke and death to be about three times higher [1]. Trials should not include centres that do not have the competence to treat patients safely, but selection should not be so exclusive that the results cannot be generalised to routine clinical practice.

### Selection and Exclusion of Patients

Concern is often expressed about highly selective trial eligibility criteria, but there are often several earlier stages of selection that are rarely recorded or reported but which can be more problematic. For example, consider a trial of a new blood pressure–lowering drug, which like most such trials is performed in a hospital clinic. Fewer than 10% of patients with hypertension are managed in hospital clinics, and this group will differ from those managed in primary care. Moreover, only one of the ten physicians who see hypertensive patients in this particular hospital is taking part in the trial, and this physician mainly sees young patients with resistant hypertension. Thus, even before any consideration of eligibility or exclusion criteria, potential recruits are already very unrepresentative of patients in the local community. It is essential therefore that, where possible, trials record and report the pathways to recruitment.

Patients are then further selected according to trial eligibility criteria. Some RCTs exclude women and many exclude the elderly and/or patients with common comorbidities. One review of 214 drug trials in acute myocardial infarction (MI) found that over 60% excluded patients aged over 75 years [6], despite the fact that over 50% of MIs occur in this older age group. A review of 41 United States National Institutes of Health RCTs found an average exclusion rate of 73% [7], but rates can be much higher. One study of the eligibility criteria of an acute stroke treatment trial found that of the small proportion of patients admitted to hospital in time to be suitable for treatment, 96% were ineligible based on the various other exclusion criteria [8]. One centre in another acute stroke trial had to screen 192 patients over two years to find an eligible patient [9]. Yet, highly selective recruitment is not inevitable. The GISSI-1 trial of thrombolysis for acute MI, for example, recruited 90% of patients admitted within 12 hours of the event with a definite diagnosis and no contraindications [10].

Strict eligibility criteria can limit the external validity of RCTs, but physicians should at least be able to select similar patients for treatment in routine practice. Unfortunately, however, reporting of trial eligibility criteria is frequently inadequate. A review of trials leading to clinical alerts by the US National Institutes of Health revealed that of an average of 31 eligibility criteria, only 63% were published in the main trial report and only 19% in the clinical alert [11]. Inadequate reporting is also a major problem in secondary publications, such as systematic reviews and clinical guidelines, where the need for a succinct message does not usually allow detailed consideration of the eligibility and exclusion criteria or other determinants of external validity.

Prerandomisation run-in periods are also often used to select or exclude patients. In a placebo run-in, all eligible patients receive placebo, and those who are poorly compliant are excluded. There can be good reasons for doing this, but high rates of exclusion will reduce external validity. Active treatment run-in periods in which patients who have adverse events or show signs that treatment may be ineffective are excluded are more likely to undermine external validity. For example, two RCTs of carvedilol, a vasodilatory beta-blocker, in chronic heart failure excluded 6% and 9% of eligible patients in treatment run-in periods—mainly because of worsening heart failure and other adverse events, some of which were fatal [1]. In both trials, the complication rates in the subsequent randomised phase were much lower than in the run-in phase.

**Table 1:**
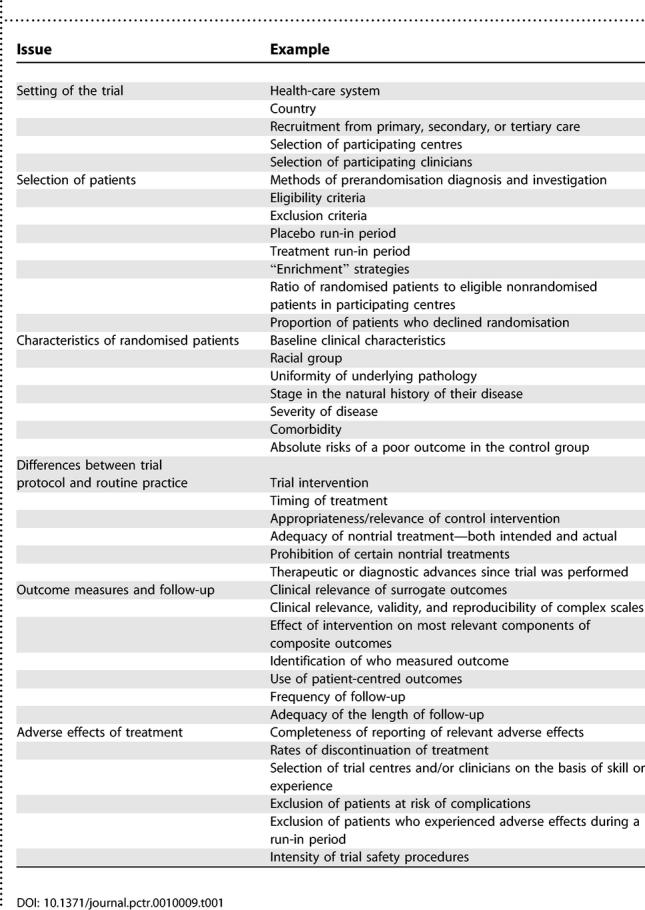
Main Issues That Can Affect External Validity and Should Be Addressed in Reports of the Results of Randomised Controlled Trials or Systematic Reviews and Considered by Clinicians

Trials also sometimes actively recruit patients who are likely to respond well to treatment (often termed “enrichment”). For example, some trials of antipsychotic drugs have selectively recruited patients who have previously had a good response to antipsychotics [1]. Other trials have excluded nonresponders in a run-in phase. One RCT of a cholinesterase inhibitor, tacrine, in Alzheimer disease recruited 632 patients to a six-week “enrichment” phase in which they were randomised to different doses of tacrine versus placebo [12]. After a washout period, only the 215 (34%) patients who had a measured improvement on tacrine in the “enrichment” phase were randomised to tacrine (at their best dose) versus placebo in the main phase of the trial. External validity is clearly undermined here.

### Characteristics of Randomised Patients

Even in large pragmatic trials with very few exclusion criteria, recruitment of less than 10% of potentially eligible patients in participating centres is common. Those patients who are recruited generally differ from those who are eligible but not recruited in terms of age, sex, race, severity of disease, educational status, social class, and place of residence. The outcome in patients included in RCTs is also usually better than those not in trials, often markedly so, not because of better treatment but because of a better baseline prognosis. Trial reports usually include the baseline clinical characteristics of randomised patients, so it is argued that clinicians can assess external validity by comparison with their patients. However, recorded baseline clinical characteristics often say very little about the real makeup of the trial population, and can sometimes be misleading. For example, Table 2 shows the baseline clinical characteristics of patients randomised to warfarin in two RCTs of secondary prevention of stroke [1]. In one trial, patients were in atrial fibrillation, and in the other they were in sinus rhythm, but the characteristics of the two cohorts were otherwise fairly similar. However, the risk of intracranial haemorrhage on warfarin was 19 times higher (*p* < 0.0001) in Stroke Prevention in Reversible Ischaemia Trial (SPIRIT) than in the European Atrial Fibrillation Trial (EAFT), even after adjustment for differences in baseline clinical characteristics and the intensity of anticoagulation [13]. In judging external validity, an understanding of how patients were referred, investigated, and diagnosed (i.e., their pathway to recruitment), as well as how they were subsequently selected and excluded, is often much more informative than a list of baseline characteristics.

**Table 2:**
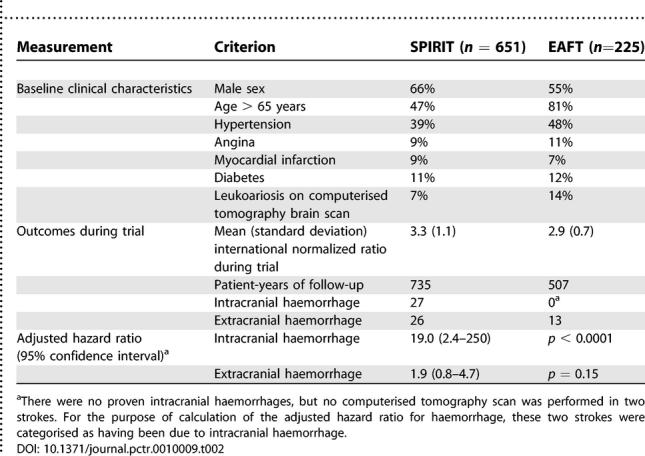
The Baseline Clinical Characteristics and Haemorrhage Outcomes of Patients Randomised to Anticoagulation with Warfarin in EAFT and SPIRIT

### The Intervention, Control Treatment, and Pre-trial or Nontrial Management

External validity can also be affected if trials have protocols that differ from usual clinical practice. For example, prior to randomisation in the RCTs of endarterectomy for symptomatic carotid stenosis, patients had to be diagnosed by a neurologist and have conventional arterial angiography, neither of which are routine in many centres. The trial intervention itself may also differ from that used in current practice, such as in the formulation and bioavailability of a drug, or the type of anaesthetic used for an operation. The same can be true of the treatment in the control group in a trial, which may use a particularly low dose of the comparator drug, or fall short of best current practice in some other way. External validity can also be undermined by too stringent limitations on the use of nontrial treatments. Any prohibition of nontrial treatments should be reported in the main trial publications, along with details of relevant nontrial treatments that were used. The timing of many interventions is also critical and should be reported when relevant.

### Outcome Measures and Follow-Up

The external validity of an RCT also depends on whether the outcomes were clinically relevant. Many trials use “surrogate” outcomes, usually biological or imaging markers that are thought to be indirect measures of the effect of treatment on clinical outcomes. However, as well as being of questionable clinical relevance, surrogate outcomes are often misleading. There are many examples of treatments that have had a major beneficial effect on a surrogate outcome, which had previously been shown to be correlated with a relevant clinical outcome in observational studies, but where the treatments have proved ineffective or harmful in subsequent large RCTs that used these same clinical outcomes [1].

Complex scales, often made up of arbitrary combinations of symptoms and clinical signs, are also problematic. A review of 196 RCTs in rheumatoid arthritis identified more than 70 different outcome scales [14]. More worryingly, a review of 2,000 RCTs in schizophrenia identified 640 scales—many of which were devised for the particular RCT and had no supporting data on validity or reliability, but which were more likely to show statistically significant treatment effects than established scales [15]. Moreover, the clinical meaning of apparent treatment effects (e.g., a 2.7-point mean reduction in a 100-point outcome scale made up of various symptoms and signs) is usually impossible to discern. Simple clinical outcomes usually have most external validity, but, even then, only if they reflect the priorities of patients. For example, patients with epilepsy are much more interested in the proportion of individuals rendered free of seizures in RCTs of anticonvulsants than they are in changes in mean seizure frequency. Identifying who actually measured the outcome can also be important. For example, the recorded operative risk of stroke due to carotid endarterectomy is highly dependent on whether patients were assessed by a surgeon or a neurologist [16].

Many trials combine events in their primary outcome measure. This can produce a useful measure of the overall effect of treatment on all the relevant outcomes, and it usually affords greater statistical power, but the outcome that is most important to a particular patient may be affected differently by treatment than the combined outcome. Composite outcomes also sometimes combine events of very different severity, and treatment effects can be driven by the least important outcome, which is often the most frequent. Equally problematic is the composite of definite clinical events and episodes of hospitalisation. The fact that a patient is in an RCT will probably affect the likelihood of hospitalisation, and it will certainly vary between different health-care systems.

Another major problem for the external validity of RCTs is an inadequate duration of treatment and/or follow-up. For example, although patients with refractory epilepsy or migraine require treatment for many years, most RCTs of new drugs look at the effect of treatment for only a few weeks. Whether initial response is a good predictor of long-term benefit is unknown. The same problem has been identified in RCTs in schizophrenia, with fewer than 50% of trials having greater than six-week follow-up, and only 20% following patients for longer than six months [17]. The contrast between beneficial effects of treatments in short-term RCTs and the less encouraging experience of long-term treatment in clinical practice has also been highlighted by clinicians treating patients with rheumatoid arthritis [18].

### Adverse Effects of Treatment

Reporting of adverse effects of treatment in RCTs and systematic reviews is often poor. In a review of 192 pharmaceutical trials, less then a third had adequate reporting of adverse clinical events or laboratory toxicology [19]. Treatment discontinuation rates provide some guide to tolerability, but pharmaceutical trials often use eligibility criteria and run-in periods to exclude patients who might be prone to adverse effects.

Clinicians are usually most concerned about external validity of RCTs of potentially dangerous treatments. Complications of medial interventions are a leading cause of death in developed countries. Risks can be overestimated in RCTs, particularly during the introduction of new treatments when trials are often done in patients with very severe disease, but stringent selection of patients, confinement to specialist centres, and intensive safety monitoring usually lead to lower risks than routine clinical practice. RCTs of warfarin in nonrheumatic atrial fibrillation are good examples. All trials reported benefit with warfarin, but complication rates were much lower than in routine practice, and consequent doubts about external validity are partly to blame for major underprescribing of warfarin, particularly in the elderly [1].

## CONCLUSIONS

Some trials have excellent external validity, but many do not, particularly some of those performed by the pharmaceutical industry. Yet researchers, funding agencies, ethics committees, medical journals, and governmental regulators all neglect proper consideration of external validity. Judgment is left to clinicians, but reporting of the determinants of external validity in trial publications, and particularly in secondary reports and clinical guidelines, is rarely adequate and much relevant information is never published. RCTs cannot be expected to produce results that are directly relevant to all patients and all settings, but to be externally valid they should at least be designed and reported in a way that allows clinicians to judge to whom they can reasonably be applied. A consensus is required on how the design and reporting of trials could be improved in order to achieve this aim. Agreement on a list of the most important issues that should be considered by clinicians and researchers would be a helpful first step.

## Abbreviations

EAFT: European Atrial Fibrillation Trial
MI: myocardial infarction
RCT: randomised controlled trial
SPIRIT: Stroke Prevention in Reversible Ischaemia Trial
